# Supplementary material for: Development of a computer-aided design software for the quantitative evaluation of aesthetic damage
Source: PLoS One. 2019 Dec 18;14(12):e0226322. doi: 10.1371/journal.pone.0226322 (PMC6919621; doi:10.1371/journal.pone.0226322)
Supplement: S1 Code site — 1. (ZIP) [file pone.0226322.s001.zip › [EESC_jr][Projeto]Relatorio_07_21/reports/templates_zip/gentelella-master/vendors/select2/docs/options.html]

---
layout: default
title: Options - Select2
slug: options
---

# Options

Select2 supports a wide variety of options that allow you to customize it to your needs.

{% include options/introduction.html %}
{% include options/core.html %}
{% include options/data.html %}
{% include options/selections.html %}
{% include options/dropdown.html %}
{% include options/events.html %}
{% include options/compatibility.html %}

{% include nav/options.html %}
